# Machine Learning Theory and Applications for Healthcare

**DOI:** 10.1155/2017/5263570

**Published:** 2017-09-27

**Authors:** Ashish Khare, Moongu Jeon, Ishwar K. Sethi, Benlian Xu

**Affiliations:** ^1^Department of Electronics and Communication, University of Allahabad, Allahabad, India; ^2^School of Electrical Engineering and Computer Science, Gwangju Institute of Science and Technology, Gwangju, Republic of Korea; ^3^Department of Computer Science and Engineering, School of Engineering and Computer Science, Oakland University, Rochester, MI, USA; ^4^School of Electrical and Automatic Engineering, Changshu Institute of Technology, Changshu, China

The explosive growth of health-related data presented unprecedented opportunities for improving health of a patient. Machine learning plays an essential role in healthcare field and is being increasingly applied to healthcare, including medical image segmentation, image registration, multimodal image fusion, computer-aided diagnosis, image-guided therapy, image annotation, and image database retrieval, where failure could be fatal.

The purpose of this special issue is to advance scientific research in the broad field of machine learning in healthcare, with focuses on theory, applications, recent challenges, and cutting-edge techniques.

The quality level of the submissions for this special issue was very high. A total of 24 manuscripts were submitted to this issue in response to the call for papers. Based on a rigorous review process, 8 papers (33%) were accepted for the publication in the special issue. Below, we briefly summarize the highlights of each paper.

One of the papers of this special issue, “Diagnosis of Alzheimer's Disease Based on Structural MRI Images Using a Regularized Extreme Learning Machine and PCA Features,” R. K. Lama et al. proposes a method and compared Alzheimer disease (AD) diagnosis approaches using structural magnetic resonance (sMR) images to discriminate AD, mild cognitive impairment, and healthy control subjects using a support vector machine (SVM), an import vector machine (IVM), and a regularized extreme learning machine (RELM). By means of experiments on the ADNI datasets, it has been concluded that RELM with the feature selection approach can significantly improve classification accuracy of AD from mild cognitive impairment and healthy control subjects.

S. Alam et al. presented a method for distinguishing AD from healthy control using combination of dual-tree complex wavelet transforms, principal coefficients from the transaxial slices of MRI images, linear discriminant analysis, and twin support vector machine in their article “Twin SVM-Based Classification of Alzheimer's Disease Using Complex Dual-Tree Wavelet Principal Coefficients and LDA.”

A semisupervised learning approach for cell detection is presented in the third article of this special issue by N. Ramesh et al. in their paper entitled “Cell Detection Using Extremal Regions in a Semisupervised Learning Framework.” The method requires very few examples of cells with simple dot annotations for training.

In the paper entitled “Patient-Specific Deep Architectural Model for ECG Classification” by K. Luo et al., a method for ECG classification is proposed. The method is based on time-frequency representation and patient-specific deep learning architectural model, and it uses deep neural network classifier.

An automatic method for segmentation of 3D magnetic resonance imaging (MRI) data, useful in the clinical diagnosis of brain tumor, named as Glioma is presented by Z. Li et al. in the article “Low-Grade Glioma Segmentation Based on CNN with Fully Connected CRF.” The method combined a multipathway convolutional neural network (CNN) and fully connected conditional random field (CRF). Experimental results have shown that the method is useful for low-grade glioma.

A general system for hybrid disease diagnosis adopting classifier optimization procedure using evolutionary algorithms is presented by M. R. Nalluri et al. in their article “Hybrid Disease Diagnosis Using Multiobjective Optimization with Evolutionary Parameter Optimization.”

Y. Chou et al., in their paper “A Real-Time Analysis Method for Pulse Rate Variability Based on Improved Basic Scale Entropy,” proposed a method named sliding window iterative base scale entropy analysis by combining base scale entropy analysis and sliding window iterative theory for analyzing heart rate variability signal.

Another paper of this special issue by J.-S. Park et al. titled “R Peak Detection Method Using Wavelet Transform and Modified Shannon Energy Envelope” presents an R peak detection method using the wavelet transform and a modified Shannon energy envelope for rapid ECG analysis.

These 8 selected contributions basically can reflect the new achievements in the machine learning applications in healthcare, and we hope they can provide a solid foundation for future new approaches and applications.

